# Use of an electronic medical record system to improve antimicrobial stewardship

**DOI:** 10.1186/cc14189

**Published:** 2015-03-16

**Authors:** P Allan, M Newman, J Collinson, L Bond, W English

**Affiliations:** 1 Royal Cornwall Hospital NHS Trust, UK

## Introduction

Antimicrobial resistance constitutes a growing global threat, driven in part by inappropriate antimicrobial prescribing [1]. Most hospitals implement antibiotic policies to promote antimicrobial stewardship. This audit examined the Royal Cornwall Hospital Trust (RCHT) Critical Care Department's compliance with the current standard defined in our local antimicrobial policy. This states that all antimicrobial prescriptions are to have an indication and review date recorded [2]. Sequential strategies to improve compliance were introduced prior to re-auditing the effects.

## Methods

The RCHT Critical Care Department utilizes the Phillips Care Vue electronic patient record. Data from this system were interrogated at three stages to assess our compliance with the trust's antimicrobial policy. The first data interrogation was performed prior to any intervention, and reflected baseline antimicrobial prescribing habits. The second data interrogation was performed during a period of active antibiotic stewardship promotion. The third data interrogation was performed following the addition of a care bundle to the prescribing module of Care Vue. This daily tick-box prompt reminded clinicians to check that all antimicrobial prescriptions had an indication and review date recorded. The records of all of the patients admitted to the critical care department during the periods of data interrogations were assessed for antimicrobial indication and review date transcription.

## Results

From the first data interrogation, antimicrobial prescriptions had an indication and review date transcription in 57% and 60% of cases respectively. Following the awareness campaign, the indication and review date transcription rate increased to 78% and 85% respectively. A daily electronic prompt was then added to our care bundle list. The final data interrogation, performed after this intervention, demonstrated that the transcription rates for both the indication and the review date had increased to 96%.

## Conclusion

We have demonstrated that the use of a daily prompt within an electronic patient record can greatly improve compliance in recording the indication and review date for all antimicrobials. These data support the widespread implementation of an electronic prescribing system where daily reminders are integrated in an effort to improve compliance with antimicrobial stewardship.
